# Short-term effects of Finnish sauna bathing on blood-based markers of cardiovascular function in non-naive sauna users

**DOI:** 10.1007/s00380-018-1202-9

**Published:** 2018-07-03

**Authors:** Setor K. Kunutsor, Arja Häkkinen, Francesco Zaccardi, Tanjaniina Laukkanen, Earric Lee, Peter Willeit, Hassan Khan, Jari A. Laukkanen

**Affiliations:** 1 National Institute for Health Research Bristol Biomedical Research Centre, University Hospitals Bristol NHS Foundation Trust, University of Bristol, Bristol, UK; 2 Musculoskeletal Research Unit, Translational Health Sciences, Bristol Medical School, University of Bristol, Learning & Research Building (Level 1), Southmead Hospital, Bristol, BS10 5NB UK; 3 Faculty of Sport and Health Sciences, University of Jyväskylä, Jyvaskyla, Finland; 4 Diabetes Research Centre, Leicester General Hospital, University of Leicester, Leicester, UK; 5 Institute of Public Health and Clinical Nutrition, University of Eastern Finland, P.O. Box 1627, 70211 Kuopio, Finland; 6 Department of Public Health and Primary Care, University of Cambridge, Cambridge, UK; 7 Department of Neurology, Medical University Innsbruck, Innsbruck, Austria; 8 Division of Cardiology, Department of Medicine, Emory University, Atlanta, GA USA; 9 Department of Internal Medicine, Central Finland Health Care District, Jyvaskyla, Finland

**Keywords:** Sauna bathing, Experimental study, Cardiovascular disease, Biomarkers

## Abstract

Emerging evidence suggests that sauna bathing is associated with reduced risk of cardiovascular and all-cause mortality events. However, the biochemical pathways by which sauna bathing might confer its effects on cardiovascular function are not certain. We aimed to study the acute effects of Finnish sauna bathing on various blood-based cardiovascular biomarkers. The study included 102 non-naive sauna users (54% male) with mean age of 51.9 years, who had at least one cardiovascular risk factor. Participants underwent a 30-min single sauna session (mean temperature, 73 °C). Biochemical profiling was conducted before, immediately after sauna and 30-min post-sauna. Overall median N-terminal pro-B-type natriuretic peptide (NT-proBNP) level (*n *= 20 participants) was 46.0 ng/L before sauna exposure, which increased to 50.5 ng/l immediately after sauna (median change, + 12.00%; *p *< 0.001) and remained persistent at 30-min post-sauna (median change from pre-sauna to post-30-min sauna, + 13.93%; *p *< 0.001). The changes were more evident in males compared with females. There were no significant changes in overall levels of high sensitivity C-reactive protein, creatine kinase, high sensitivity troponin I, and creatine kinase-MBm. However, levels of creatine kinase increased in males (median change immediately after sauna, + 2.99%; *p *= 0.024). Levels of NT-proBNP increased after sauna exposure. The increase in levels of creatine kinase was more evident in males. Long-term interventional studies are warranted to evaluate if these biomarkers are involved in pathways underlying the associations of sauna bathing with cardiovascular outcomes.

## Introduction

Repeated passive heat therapy has been shown to have beneficial effects on the cardiovascular system by improving endothelial function, arterial stiffness, and microvascular function; reducing carotid intima media thickness, blood pressure, markers of oxidative stress, and brain natriuretic peptides; as well as increasing parasympathetic and decreasing sympathetic nervous activity [1–5]. Sauna bathing, a form of passive heat therapy, is an activity which is a tradition in Finland and commonly used for relaxation and pleasure and is becoming increasingly popular in many other cultures [6, 7]. Finnish sauna bathing has been linked to improved cardiovascular function as well as several health benefits. Emerging evidence suggests that regular sauna bathing is associated with lower blood pressure [8], reduced risk of cardiovascular disease (CVD) mortality [9], stroke [10], dementia [11], and pulmonary diseases [12, 13], as well as reduced all-cause mortality risk [9]. The specific cardiovascular adaptations produced by long-term sauna bathing is not certain, but the effects of sauna bathing on cardiometabolic health outcomes have been linked to its beneficial impact on circulatory and cardiovascular function. Regular long-term sauna bathing has been shown to produce blood pressure lowering effects [14] as well as reducing levels of markers of inflammation [15, 16].

Evidence on the beneficial effects of sauna bathing on circulatory and cardiovascular function has mostly been based on studies evaluating the short-term effects of sauna. Short-term or acute sauna exposure has been shown to produce blood pressure lowering effects [17], decrease peripheral vascular resistance [17, 18] and arterial stiffness [8, 19] as well as improve arterial compliance [19]. Short-term sauna exposure also activates the sympathetic nervous system and the renin–angiotensin–aldosterone system, and the hypothalamus–pituitary–adrenal hormonal axis with short-term increases in levels of their associated hormones [20]. Other potential pathways by which sauna therapy may exert its cardioprotective effects are via positive modulation in levels of blood-based established cardiovascular risk factors such as lipids, glucose, markers of insulin resistance, natriuretic peptides, and cardiac troponins. There is, however, limited evidence on the effects of short-term sauna exposure on these circulating biomarkers. Several studies on the short-term effects of other passive heat therapies such as Waon therapy, infrared saunas, intermittent warm water immersion, and whole-body hyperthermia have, however, been shown to have beneficial effects on parameters of cardiovascular function such as (1) reductions in concentrations of circulating arterial endothelial- and platelet-derived microparticles [21] and markers of inflammation and oxidative stress [3, 22–25]; (2) protection against impaired vascular function [26]; (3) induction in systemic and hemodynamic responses (blood pressure lowering and shear rate increases) [27, 28] and improved vascular function [29]; and (4) reduction in arterial stiffness [30].

Given the overall evidence which suggests that short-term heat therapy may produce widespread beneficial effects on vascular function, our hypothesis was that a short-term exposure to Finnish sauna will produce beneficial changes in levels of circulating cardiovascular biomarkers. In this context, we conducted this exploratory experimental non-controlled study to show the effects of 30 min of Finnish sauna bathing on several parameters of cardiovascular function. We have previously reported on the potential beneficial effects of acute sauna exposure on arterial stiffness and compliance, blood pressure, haematological variables, as well as renal markers [8, 19]. In this report, we study the effects of 30 min of Finnish sauna bathing on circulating blood-based markers of cardiovascular function [such as high sensitivity C-reactive protein (hsCRP), creatine kinase, hs-troponin I, creatine kinase-MBm, and N-terminal pro-B-type natriuretic peptide (NT-proBNP)], immediately after sauna and 30-min post-sauna.

## Materials and methods

### Participants

We recruited 110 participants without pre-existing CVD from the city of Jyväskylä, Central Finland region, through the local out-of-hospital community health care center. Study participants were asymptomatic with at least one cardiovascular risk factor, such as a history of smoking, dyslipidaemia, hypertension, obesity, diabetes, or family history of coronary heart disease (CHD). Participants (*n *= 8) with any form of acute or pre-existing CVD were excluded from the study. A total of 102 participants were finally included in the study. Given the challenge in recruiting naive or non-sauna users to sauna studies in Finland, because the majority of the population use sauna regularly, our study sample were all sauna users. Prior to study entry, participants were provided with information about the research purposes and measurement procedures and were screened by a cardiac specialist. The study design and protocol were approved by the institutional review board of the Central Finland Hospital District ethical committee, Jyväskylä, Finland (Dnro 5U/2016). All study participants provided written informed consent.

### Baseline assessments and clinical examination

Baseline data collection, as well as a clinical evaluation, was conducted on separate days prior to the experiment. Baseline assessments and sauna measurements were conducted during June and November 2016. Assessment of demographics, lifestyle factors (e.g., smoking, physical activity, and sauna bathing habits), prevalent diseases, and regular use of medication were based on a detailed self-reported questionnaire which was checked by a cardiologist during screening. Physical activity and sauna bathing habits over the past 1 year were assessed. The duration and frequency of physical activity were also explored. Information collected on regular sauna bathing habits was based on frequency (weekly sauna sessions), duration, and temperature (measured using a thermometer located in the sauna room). Medical history, physical examination, and resting electrocardiogram (ECG) were assessed during the screening visit. Resting blood pressure was estimated as the mean of two measurements obtained, while the participant was in the supine position, based on a standardized measurement protocol. Body mass index (BMI) was calculated by dividing weight in kilograms by the square of height in meters.

### Finnish sauna exposure

The traditional Finnish sauna with dry air and relatively high temperature was used as our exposure [9]. It consisted of a typical Finnish sauna bathing session which lasted for 30 min. However, there was a short, 2-min shower after the first 15 min [8]. Sauna bathing sessions took place between 9.00 and 11.00 h on the specified study days. Sauna rooms were gender specific and all participants wore their own swim suits during the sauna sessions. Only one participant was allowed in the sauna bath at a time. Sauna temperature was set at 80 °C and this was controlled and monitored by internal temperature sensors designed by Harvia Oy, Finland. Temperature assessment was continuous with the use of a two-channel thermometer in the sauna room and the data were collected during experiment. The temperature sensor also monitored the humidity of the sauna room. Based on overall data collected, the mean ± standard deviation (SD) temperature was 73 ± 2 °C with a relative humidity of 10–20%. Study participants were monitored and supervised by a physician and were allowed to leave the sauna at any time they felt any discomfort. All participants underwent the recommended sauna protocol without any problems. Participants were given 500 mL of still water at room temperature to drink during the entire sauna session, and including the recovery period post-sauna. The recovery period was for 30 min which involved resting in a designated relaxing waiting lounge (mean temperature 21 °C). Body temperature was measured for each participant from the ear.

### Assessment of laboratory markers

Non-fasting blood samples were taken about 2 h prior to sauna sessions. Participants were instructed to abstain from strenuous physical activity 24 h before the blood samples were taken. Venous blood samples were collected by a qualified laboratory technician from the antecubital vein in the sitting position, using sterile needles, and were collected into serum and plasma tubes (BD Vacutainer, Plymouth. UK). Whole blood samples were stored for 10 min before being centrifuged at 3500 rpm (Megafuge, Heraeus, Germany) and serum and plasma samples stored at − 80 °C until analysis. Serum total cholesterol, high-density lipoprotein (HDL), low-density lipoprotein (LDL), Apolipoprotein A1 (ApoA1), Apolipoprotein B (Apo B), triglycerides, and plasma glucose concentrations were measured using a spectrophotometry analyzer (Konelab 20XTi, Thermo Fisher Scientific, Vantaa, Finland). Venous blood samples for the determination of plasma inflammatory markers and cardiac biomarkers were analyzed using chemiluminecent immunoassay by Siemens Immulite 2000 XPi (Siemens Healthcare Diagnostics Products Ltd., Llanberies, UK) analyzer. Measurements of NT-proBNP were conducted in only a random sample of participants because of the high costs associated with these assays. Creatine kinase was measured in heparinised plasma by Konelab 20XTi, (Thermo Fisher Scientific, Vantaa, Finland). All biomarkers were assessed before and after sauna.

### Statistics

All the cardiovascular biomarkers (hsCRP, creatine kinase, hs-troponin I, creatine kinase-MBm, and NT-proBNP) evaluated were skewed and thus were log transformed to approximate normal distributions. Data are presented as means (SD) or median (interquartile range, IQR) for continuous variables according to their distribution and as proportions for categorical variables. Normality was checked using the Shapiro–Wilk test as well as through observing the Q–Q plots. Repeated measure analysis of variance (ANOVA) was used to estimate within-group differences and changes by time. Paired *t* tests were used to compare within-group changes in biomarkers assessed immediately after sauna and 30-min post-sauna. Given the skewness of cardiovascular biomarkers, median percent changes were calculated as they are robust and provide strong reductions in estimation bias and variance in the presence of outliers [31]. A two-sided significance level was set at *p *≤ 0.05. All statistical analyses were carried out with Stata version 14.1 (Stata Corp, College Station, Texas, USA).

## Results

### Characteristics of population

Baseline characteristics of the 102 study participants are shown in Table 1. The majority of participants were male (*n *= 56). The mean (SD) of age, BMI, and SBP were 51.9 (SD 9.2) years, 27.9 (4.7) kg/m^2^, and 136 (16) mmHg, respectively. Current smokers made up 14.4% of the study population. Lipid parameters were generally similar between males and females. Male participants had higher levels of creatine kinase, creatine kinase-MBm, and NT-proBNP compared with females. Levels of hsCRP and hs-troponin I were higher in female. Levels of these cardiovascular risk markers in study participants were generally within the normal reference ranges. Mean plasma creatinine level was 114.5 U/L in this population. Dyslipidaemia (63.0%) and family history of CHD (34.0%) were the most common cardiovascular risk factors in the study population, followed by hypertension at 14.3%. Forty-four percent of subjects had both dyslipidaemia and a family history of CHD. A few participants had underlying clinical conditions such as type 1 diabetes (2.0%), type 2 diabetes (1.0%), respiratory diseases (5.1%), thyroid disease (3.1%), skin disease (4.0%), and rheumatoid arthritis (1.0%). Baseline assessment of sauna bathing showed that the majority of participants used sauna baths 3 times per week (43.9%). Commonly, a sauna session lasted between 20 and 40 min for most participants (56.7%) and the average self-reported temperature was 72 °C. Most participants reported > 1–3 sessions of physical activity per week (32.3%) followed by > 3–5 sessions per week (30.6%). The majority of participants (37.1%) spent > 60 min during a typical physical activity session.

**Table 1:** Baseline characteristics

	*N* (*M*/*F*)	Total	*M*	*F*
Clinical characteristics				
Age (years)	56/46	51.9 ± 9.2	51.8 ± 10.1	52.0 ± 8.3
Body weight (kg)	56/46	82.7 ± 16.0	88.9 ± 13.4	75.5 ± 15.9
Body mass index (kg/m^2^)	56/46	27.2 (24.5–30.7)	27.7 (25.1–30.6)	26.7 (23.8–31.0)
Smokers	56/46	14 (14.4%)	9 (17.7%)	5 (10.9%)
Systolic blood pressure (mmHg)	56/46	136.5 ± 16.2	137.2 ± 14.4	135.8 ± 18.3
Diastolic blood pressure (mmHg)	56/46	82.1 ± 9.6	84.2 ± 9.6	79.6 ± 9.2
Resting HR (bpm)	56/46	64 (59–70)	64 (56.5–70)	65 (61–70)
Total cholesterol (Chol_tot_; mmol/L)	56/46	5.4 ± 1.0	5.4 ± 1.1	5.4 ± 0.9
Low-density lipoprotein (LDL; mmol/L)	56/46	3.0 ± 0.8	3.1 ± 0.8	3.0 ± 0.8
High-density lipoprotein (HDL; mmol/L)	56/46	1.4 ± 0.4	1.3 ± 0.4	1.5 ± 0.4
Triglycerides (mmol/L)	56/46	1.6 (1.0–2.3)	1.8 (1.2–2.6)	1.3 (0.9–2.0)
Glucose (mmol/L)	56/46	5.2 ± 0.9	5.3 ± 1.0	5 ± 0.9
Apo A1 (g/L)	56/46	1.5 (1.3–1.7)	1.5 (1.3–1.7)	1.6 (1.4–1.8)
Apo B (g/L)	56/46	1.0 (0.8–1.2)	1.0 (0.8–1.2)	0.9 (0.7–1.1)
Cardiovascular risk markers				
High sensitivity C-reactive protein (mg/L) (mg/L)	54/46	1.26 (0.52–2.79)	1.12 (0.59–3.31)	1.39 (0.48–2.51)
Creatine kinase (U/L)	54/46	114.5 (81.0–160.0)	132.5 (100.0–189.0)	99.5 (71.0–122.0)
High sensitivity troponin I (ng/mL)	54/46	0.003 (0.001–0.015)	0.002 (0.001–0.014)	0.005 (0.001–0.015)
Creatine kinase-MBm (ng/mL)	54/46	1.39 ( 0.99–2.14)	1.90 (1.34–2.67)	1.18 (0.71–1.42)
N-terminal pro-B-type natriuretic peptide (ng/L)	8/12	46.0 (32.5–58.0)	48.0 (25.5–53.5)	44.0 (34.5–74.5)
Self-reported conditions and family history				
Hypertension	56/46	14 (14.3%)	11 (21.2%)	3 (6.5%)
Hypercholesterolaemia^a^	56/44	63 (63.0%)	37 (66.1%)	26 (59.1%)
Type 1 diabetes	56/46	2 (2.0%)	1 (1.9%)	1 (2.2%)
Type 2 diabetes	56/46	1 (1.0%)	1 (1.9%)	0 (0.0%)
Respiratory (incl. Asthma)	56/46	5 (5.1%)	2 (3.8%)	3 (6.5%)
Thyroid disease (hypo)	56/46	3 (3.1%)	1 (1.9%)	2 (4.3%)
Skin disease (atopic)	56/46	4 (4.1%)	3 (5.8%)	1 (2.2%)
Rheumatoid arthritis	56/46	1 (1.0%)	0 (0.0%)	1 (2.2%)
Family history of coronary heart disease	56/46	33 (34.0%)	15 (29.4%)	18 (39.1%)
Self-reported sauna habits and physical activity				
Frequency of sauna, times/week	56/46			
1		16 (16.3%)	6 (11.5%)	10 (21.7%)
2		16 (16.3%)	7 (13.5%)	9 (19.6%)
3		43 (43.9%)	23 (44.2%)	20 (43.5%)
4		23 (23.5%)	16 (30.8%)	7 (15.2%)
Duration of sauna, minutes/session	56/45			
< 20		17 (17.5%)	8 (15.4%)	9 (20.0%)
20–40		55 (56.7%)	28 (53.8%)	27 (60.0%)
41–60		21 (21.6%)	14 (26.9%)	7 (15.6%)
> 60		4 (4.1%)	2 (3.8%)	2 (4.4%)
Average temperature of sauna, °C	56/46	72.0 ± 8.5	73.0 ± 9.0	70.9 ± 7.8
Frequency of physical activity, times/week	56/46			
0		1 (1.6%)	1 (3.3%)	0 (0.0%)
1		16 (25.8%)	7 (23.3%)	9 (28.1%)
2–3		20 (32.3%)	11 (36.7%)	9 (28.1%)
4–5		19 (30.6%)	9 (30.0%)	10 (31.3%)
> 5		6 (9.7%)	2 (6.7%)	4 (12.5%)
Duration of physical activity, minutes/session	56/46			
<20		7 (10.0%)	2 (5.7%)	5 (14.3%)
20–40		18 (25.7%)	12 (34.3%)	6 (17.1%)
41–60		19 (27.1%)	11 (31.4%)	8 (22.9%)
> 60		26 (37.1%)	10 (28.6%)	16 (45.7%)

Values are reported as mean ± SD or median (interquartile range) for continuous variables and *n* (%) for categorical variables

*M* male, *F* female

^a^Total Cholesterol > 5.17 mmol/L

### Sauna and changes in cardiovascular biomarkers

**Fig. 1:**
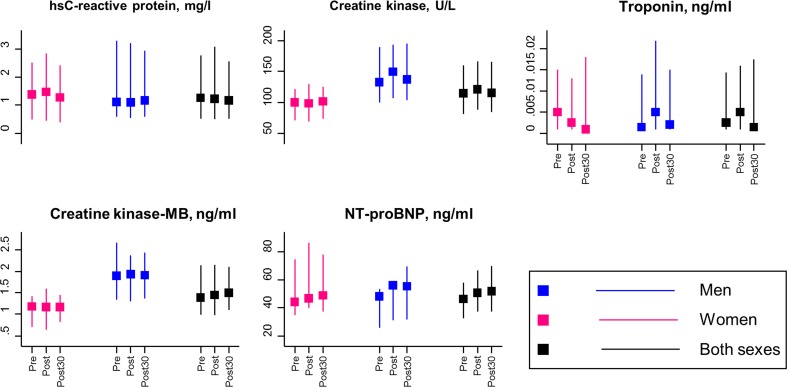
Levels of cardiovascular biomarkers at pre-sauna, immediately after sauna, and 30 min after sauna. Data are reported as medians (interquartile ranges)

Table 2 and Fig. 1 show levels of evaluated cardiovascular biomarkers at pre-sauna, immediately after sauna, and 30 min after sauna. Overall NT-proBNP levels were 46.0 ng/L before sauna exposure, which increased to 50.5 ng/L immediately after sauna (median percentage change, 12.00%; *p *< 0.001). Increased levels of NT-proBNP remained persistent at 30-min post-sauna, 51.5 ng/L (median percentage change from pre-sauna to post-30-min sauna, 13.93%; *p *< 0.001) (Table 3). These changes were more evident in males compared with females. Overall, there were no statistically significant changes in levels of hsCRP, creatine kinase, hs-troponin I, and creatine kinase-MBm immediately after sauna and 30 min after sauna. However, levels of creatine kinase increased immediately after sauna in males; from 132.5 to 149.5 U/L (median relative change, 2.99%; *p *= 0.024). Levels of hs-troponin I seemed to increase immediately after sauna in males, but this change was very small and marginally significant (0.002–0.005 ng/mL; *p *= 0.058) (Table 3).

**Table 2:** Levels of cardiovascular biomarkers before and after sauna

	*N* (M/W)	Total	*M*	*F*
High sensitivity CRP (mg/L)				
Pre	54/46	1.26 (0.52–2.79)	1.12 (0.59–3.31)	1.39 (0.48–2.51)
Post	53/46	1.22 (0.51–3.08)	1.09 (0.57–3.21)	1.48 (0.45–2.86)
Post 30 min	51/38	1.17 (0.53–2.57)	1.17 (0.59–2.94)	1.28 (0.40–2.42)
*p* value*		0.852	0.768	0.535
Creatine kinase (U/L)				
Pre	54/46	114.5 (81.0–160.0)	132.5 (100.0–189.0)	99.5 (71.0–122.0)
Post	54/46	121.0 (88.0–166.0)	149.5 (107.0–193.0)	98.0 (69.0–130.0)
Post 30 min	50/38	115.5 (84.5–165.5)	137.0 (104.0–195.0)	101.0 (74.0–125.0)
*p* value*		0.037	0.027	0.059
High sensitivity troponin I (ng/mL)				
Pre	54/46	0.003 (0.001–0.015)	0.002 (0.001–0.014)	0.005 (0.001–0.015)
Post	53/46	0.005 (0.001–0.016)	0.005 (0.001–0.022)	0.003 (0.001–0.013)
Post 30 min	50/38	0.002 (0.001–0.018)	0.002 (0.001–0.015)	0.001 (0.001–0.018)
*p* value*		0.331	0.145	0.736
Creatine kinase-MBm (ng/mL)				
Pre	54/46	1.39 (0.99–2.14)	1.90 (1.34–2.67)	1.18 (0.71–1.42)
Post	53/46	1.45 (0.97–2.15)	1.93 (1.31–2.38)	1.17 (0.64–1.60)
Post 30 min	51/38	1.50 (1.09–2.10)	1.91 (1.36–2.43)	1.17 (0.82–1.45)
*p* value*		0.296	0.450	0.562
NT-proBNP (ng/L)				
Pre	8/12	46.0 (32.5–58.0)	48.0 (25.5–53.5)	44.0 (34.5–74.5)
Post	8/12	50.5 (37.0–66.5)	56.0 (31.0–59.0)	46.5 (39.5–86.5)
Post 30 min	8/12	51.5 (37.0–70.0)	55.0 (31.5–69.5)	48.5 (37.0–78.0)
*p* value*		<0.0001	<0.0001	0.066

*CRP* C-reactive protein, *NT-proBNP* N-terminal pro-B-type natriuretic peptide

*p value for anova; all values are reported as median (interquartile range)

**Table 3:** Median percentage changes in cardiovascular biomarkers

	Total	*M*	*F*
High sensitivity CRP (mg/L)			
Post	1.71 (− 0.38, 5.28)	5.13 (1.03, 9.49)	− 0.80 (− 3.98, 2.79)
Post 30 min	0.62 (− 1.40, 2.58)	3.06 (− 1.09, 5.45)	− 0.89 (− 2.06, 1.79)
*p* value (post versus pre)	0.635	0.607	0.927
*p* value (post 30 versus pre)	0.532	0.345	0.239
Creatine kinase (U/L)			
Post	1.82 (− 0.96, 3.34)	2.99 (1.66, 4.76)	− 1.61 (− 3.02, 2.78)
Post 30 min	− 0.90 (− 2.70, 1.47)	0.00 (− 1.38, 2.40)	− 2.70 (− 5.10, 1.68)
*p* value (post versus pre)	0.085	0.024	0.511
*p* value (post 30 versus pre)	0.832	0.601	0.115
High sensitivity troponin (ng/mL)			
Post	0.00 (0.00, 0.00)	0.00 (0.00, 1.18)	0.00 (0.00, 5.26)
Post 30 min	0.00 (0.00, 0.00)	0.00 (0.00, 0.00)	0.00 (− 18.61, 0.00)
*p* value (post versus pre)	0.269	0.058	0.788
*p* value (post 30 versus pre)	0.576	0.779	0.296
Creatine kinase-MBm (ng/mL)			
Post	1.68 (− 4.77, 8.70)	− 0.51 (− 5.86, 8.29)	3.30 (− 7.18, 13.06)
Post 30 min	− 0.84 (− 5.86, 8.61)	− 2.51 (− 7.77, 7.79)	0.51 (− 9.54, 25.86)
*p* value (post versus pre)	0.453	0.632	0.526
*p* value (post 30 versus pre)	0.128	0.351	0.236
NT-proBNP (ng/L)			
Post	12.00 (9.44, 17.70)	17.57 (9.38, 28.35)	9.79 (0.08, 14.14)
Post 30 min	13.93 (7.61, 29.66)	26.42 (11.78, 41.90)	8.19 (− 5.50, 18.01)
*p* value (post versus pre)	< 0.001	< 0.001	0.077
*p* value (post 30 versus pre)	< 0.001	< 0.001	0.073

Values are reported as median (95% confidence intervals); *p* values are from paired *T* tests based on the log transformed variables

*CRP* C-reactive protein, *NT-proBNP* N-terminal pro-B-type natriuretic peptide

## Discussion

In this first experimental study to assess the acute effects of Finnish sauna exposure on specific blood-based cardiovascular biomarkers, we have shown that levels of cardiac markers such as NT-proBNP and creatine kinase increase immediately after 30 min of exposure to sauna. The increase in levels was more pronounced for NT-proBNP and these elevated levels seem to be sustained 30 min after the sauna exposure. The changes in biomarkers such as creatine kinase were generally more evident in males. There were no statistically significant changes in levels of hsCRP and creatine kinase-MBm both immediately after sauna and post-30-min sauna; though there was a suggestion of an increase in levels of hs-troponin I immediately after sauna in males, but this change was very negligible and marginally significant.

Emerging data suggest that long term or repeated exposure to sauna bathing is associated with reduced risk of cardiovascular outcomes such as hypertension [14, 32], stroke, cardiovascular, and overall mortality [9]. Studies of both Finnish sauna bathing and other passive heat therapies suggest that the protective effects of thermal therapy on these outcomes might be mediated via their beneficial effects on blood pressure, endothelial function, microvascular function, arterial stiffness, vascular resistance, and carotid intima media thickness [1–5, 8, 17–19]. Since levels of blood-based cardiovascular markers such as CRP, creatine kinase, troponin, NT-proBNP, markers of inflammation and oxidative stress, and lipids have been linked to cardiovascular outcomes such as CHD, stroke, and heart failure [33–36], it is biologically plausible that the effects of sauna bathing on cardiovascular function might be mediated through beneficial changes in these cardiac-related biomarkers. Indeed, in two studies that evaluated the effect of Finnish sauna bathing on lipid profile, there were significant decreases in total cholesterol and LDL cholesterol after 2–3 weeks of sauna exposure [37, 38]. One study suggested that the lipid changes as a result of sauna exposure were similar to that produced by moderate-intensity physical exercise [37]. We have also previously shown that long-term sauna exposure is associated with reduction in levels of markers of inflammation [15, 16]. However, some of the mechanistic pathways implicated for the cardioprotective effects of sauna bathing did not appear to be consistent with our current findings, as we observed only minor changes due to a single sauna session. It is difficult to compare our results in the context of the previous studies, as this is the first study to evaluate the acute effects of a traditional Finnish sauna exposure on circulating levels of these particular cardiovascular risk markers. However, a previously published study demonstrated a delayed increase in plasma levels of atrial natriuretic peptide (ANP) after 20 min of exposure in a Finnish sauna [39].

A number of studies evaluating the effects of other passive heat therapies such as Waon therapy, infrared saunas, intermittent warm water immersion, and whole-body hyperthermia have demonstrated beneficial modulation in levels of blood-based circulating markers of cardiovascular function. While studies on the acute effects of these therapies are sparse, there are a number of studies on the repeated or long-term effects of these thermal therapies. In individuals exposed to about 56 min of passive heat stress, circulating concentrations of arterial endothelial- and platelet-derived microparticles (which may play a pathogenic role in vascular disease [40–42]) were found to be markedly reduced [21]. Repeated passive heat therapy has also been shown to reduce levels of circulating markers of inflammation and oxidative stress [3, 22–25]. Ohori et al. demonstrated that 3 weeks of repeated thermal treatment (Waon therapy) in patients with chronic heart failure was associated with improvement in levels of BNPs and plasma norepinephrine [43]. In several other studies in which patients with heart failure were treated with infrared-ray sauna therapy for several weeks, decreases in concentrations of BNPs were demonstrated [44, 45]. An essential finding in our study was the elevation in NT-proBNP levels after sauna bathing, which may be an indicator of increased workload of the cardiovascular system and heart muscle due to a single 30-min sauna exposure. There is no active function of skeletal muscles during the sauna bathing, which is in contrast to the training response experienced during physical activity. However, increased heart rate increases myocardial workload and oxygen demand similar to physical exercise. Supporting our preliminary findings, in a previous exercise-exposure study, a significant increase in NT-proBNP was observed after 10-km recreational running exercise session [46]. N-terminal pro-B-type natriuretic peptides, which are the more stable by-products of circulating B-type natriuretic peptides, are known to play a role in the regulation of blood pressure and sodium balance [47]. Above the reference range, increased concentrations of NT-proBNP have been recommended for the diagnosis and treatment guidance of heart failure [48, 49]. Recent evidence suggests that information on NT-proBNP is predictive of first-onset heart failure, CHD, and stroke [33]. Another finding in our study was a slight increase in levels of hs-troponin I in males immediately after sauna, but the difference was marginally significant. Troponin is released from the cytosolic pool of the myocytes and it may be released during prolonged ischemia with degradation of actin and myosin filaments and it has been even used as a marker of minor cardiac muscle injury [50]. Compared with the older troponin assays, hs-troponin I assays improve the speed of diagnosis and are well-suited for detecting sub-clinical cardiac structural abnormalities [50, 51]. High-sensitivity cardiac troponins have also been suggested to be prognostic biomarkers for cardiovascular events in normal- and high-risk general populations [52]. The minor changes in levels of these markers could be due to the short-term nature of the sauna exposure (only 30 min). Previous studies of other passive heat therapies with longer exposure time have demonstrated marked changes in levels of some of these circulating biomarkers [43, 44]. Long-term intervention studies are indeed warranted to study the effects of sauna bathing on these cardiac-specific biomarkers.

To our knowledge, we have conducted the first study to evaluate the acute effects of 30-min sauna exposure on emerging biomarkers for cardiovascular function, based on traditional hot and dry Finnish sauna bathing which is a different exposure compared to other passive heat therapies such as warm water immersion, Waon therapy, and infrared heat, which are characterized by lower temperatures. Given the experimental setting of the current study, the number of study subjects recruited was adequate to assess any meaningful clinical changes in indices evaluated. The intervention simulated a typical dry and hot Finnish sauna session which lasted for 30 min and no adverse events occurred duration the entire duration of the study. These observations suggest that 30 min of sauna bathing can be considered a safe activity for the cardiovascular system, findings which are consistent with reports that regular sauna bathing can be safely recommended for majority of healthy people and patients with stable heart disease [32]. A number of limitations deserve consideration. A major limitation was that study participants were not sauna-naive users or did not include non-sauna users, which could have caused biases as the intervention is not novel to study participants. This situation could not be avoided, because the majority of the Finnish population are sauna users, given that sauna bathing is embedded in the tradition. The intervention was short-term and we employed a before- and after-design without the use of a control; however, this study was designed to be a pilot study and was exploratory in nature given the novel nature of the topic. We had measurements of NT-proBNP in only a small number of participants, which was due to the costs associated with such measurements. Follow-up period of recovery was limited to a 30-min period. Due to the novel study protocol which focused on acute changes in inflammatory, muscle and cardiac biomarkers, we did not assess prolonged or long-lasting changes hours or days after sauna exposure.

## Conclusions

Our experimental study suggests that levels of NT-proBNP and creatine kinase increase after short-term sauna exposure. These changes are more evident in males. Long-term interventional studies are warranted to evaluate if these biomarkers are involved in pathways underlying the protective associations of sauna bathing with adverse cardiovascular outcomes.
